# Oxytocin Neurons Are Essential in the Social Transmission of Maternal Behavior

**DOI:** 10.3389/fnbeh.2022.847396

**Published:** 2022-03-18

**Authors:** Xin Fang, Yongjie Wang, Zhihui Huang

**Affiliations:** ^1^Department of Human Anatomy and Histoembryology, School of Basic Medical Sciences, Hangzhou Normal University, Hangzhou, China; ^2^School of Pharmacy and Department of Neurosurgery, The Affiliated Hospital, Hangzhou Normal University, Hangzhou, China; ^3^Key Laboratory of Elemene Class Anti-Cancer Chinese Medicines, Hangzhou Normal University, Hangzhou, China; ^4^Engineering Laboratory of Development and Application of Traditional Chinese Medicines, Hangzhou Normal University, Hangzhou, China; ^5^Collaborative Innovation Center of Traditional Chinese Medicines of Zhejiang Province, Hangzhou Normal University, Hangzhou, China

**Keywords:** oxytocin, social transmission, maternal behavior, alloparenting, paraventricular nucleus

## Introduction

Virgin females and males of many species generally avoid infants, feeling stimuli aversive, yet parturient mothers typically find pups irresistible and display a suite of maternal nurturing behaviors. Parental behaviors, including nursing and protection of the young, are of great importance for the survival, mental as well as the physical wellbeing of the offspring (Rilling and Young, 2014). Alloparenting, defined as caring provided by other individuals than parents, is a universal behavior among humans that has shaped our evolutionary history and remains important in contemporary society. Humans exhibit high degrees of cooperation, such as proactive food sharing, cooperative childcare teaching, cultural learning, and collaborative foraging (Jaeggi and Gurven, 2013). In human societies, alloparenting promotes consistent energetic input for infants and reduces demands on maternal time and energy allocation (Meehan, 2009; Kramer and Veile, 2018). The quality and quantity of alloparental care may act as predictive factors of social emotional and cognitive linguistic outcomes and childcare quality may interact with infant temperament to predict behavioral problems and social competence. Although there was a study about alloparenting of many species (Kenkel et al., 2017), psychophysiological understanding of alloparenting is still largely unknown (Glasper et al., 2019).

Oxytocin (OT), one of the earliest and most significant discoveries in social neuroscience, has received extensive attention both from the scientific community and the public (Marlin and Froemke, 2017). It is mainly synthesized in the paraventricular nucleus (PVN) and supraoptic nucleus of the hypothalamus and is released into the peripheral circulation from the posterior pituitary gland. In mice, OT enables neuroplasticity in the auditory cortex for maternal recognition of pup distress (Kim and Strathearn, 2016). A previous study has shown that the density of OT receptors in the nucleus accumbens of female prairie voles was positively correlated with alloparental behavior (Olazábal, 2014). Moreover, Feldman and colleagues reported that parental sensitive caregiving, including warmth, gaze duration, checking behaviors, responsiveness to child's cues, and engagement, assessed by coding interactive behavior were correlated positively with parental endogenous OT (plasma and salivary OT) in human beings (Feldman et al., 2011). Knockdown of OT receptors in this region in the juvenile period decreased subsequent alloparenting behavior while overexpression of OT increased alloparenting behavior (Keebaugh and Young, 2011). Mammals have strong learning capabilities, and skills could be transmitted from old individuals to young ones. Can maternal nurturing behavior be transmitted from experienced individuals to virgins, and is the mechanism and process associated with OT? Recently, Prof. Robert C. Froemke's team from New York University published a research article in *Nature* entitled “OT neurons enable social transmission of maternal behavior” (Carcea et al., 2021), which investigated these questions.

## Oxytocin Neurons' Roles in the Social Transmission of Maternal Behavior

To investigate the relationship between OT and alloparenting behavior, Froemke's team established a system that could combine behavioral and neural activity monitoring in cages of mother, litter and co-housed virgin female mice. The camera with visible (daytime) and infrared light (nighttime) made the uninterrupted experimental observation possible. They quantified the frequency and duration of specific behaviors by building ethograms (Dhawale et al., 2017). According to a previous report (Kenkel et al., 2017), the alloparenting behaviors such as pup retrieval, licking/grooming, and arched-back huddling, are not qualitatively different from the behavior of parents. As expected, the authors found that the virgin mice started to engage in parenting tasks after observing maternal behavior of co-housed dams. They initiated the reliable pup retrieving earlier than virgins co-housed with pups but without dams and began to spend time in the nest with pups. The study found that the dams attempted to keep virgins in the nest and encouraged them to contact pups and the intercommunication between dams and virgins were positively correlated with virgin retrieval. Furthermore, the onset of retrieval behavior decreased after PVN neurons were specifically silenced, which resulted in the blocking of OT release. Further analysis of OT neuronal activation by single-unit recordings showed that the PVN and OT-PVN units of virgins were activated during intercommunication with dams, both the spiking rate and the firing rate were upregulated in the OT neurons of PVN more obviously in virgins who were guided by dams to perform nest entry behaviors than those of voluntary entry ones.

To confirm whether the alloparenting could also be enhanced in non-co-housed virgins who could observe pup retrieval by dams, the authors performed ten retrieval tests with the dams in the presence of the virgins for four consecutive days under four different co-housed conditions, which were defined as no barrier, with a transparent barrier, with opaque barrier and with a transparent barrier but knockout oxytocin receptor (OXTR) in virgins respectively. The results showed that the transmission of retrieval behavior was successfully acquired by virgins even inserting a transparent barrier between the observing pup-naive virgins and the retrieving dams because there were similar virgin retrieving rates between the no barrier and transparent barrier group. Most virgins behind a transparent barrier became skilled in retrieving while virgins behind an opaque barrier could not, which suggested that visual input was very important for this kind of experience transmission. On the other hand, OT signaling was also necessary, because virgins did not acquire retrieving experiences through a transparent barrier after OXTR was knocked out.

As the visual input was necessary for acquiring alloparenting behavior, the authors identified visual inputs onto OT neurons by means of monosynaptic rabies-based retrograde tracing. They found that projections from medial superficial layers of ipsilateral superior colliculus to PVN were involved in visual processing. Retrievals by dams could activate PVN neurons in non-co-housed virgins during observational testing, even though the virgins were not interacting with the dams or pups. In other words, intercommunication between dams and virgins may cause somatosensory or arousal-based PVN input, and the nursing pups' behavior of dams finally led to collicular visual response.

Cortical plasticity plays an important role in neural adaptation with motherhood. In mice, pup-naive virgin females do not recognize the meaning of pup distress calls, but they can retrieve isolated pups to the nest after having been co-housed with a mother and litter. Maternal behavior comes from intrinsic mechanisms and experience-dependent plasticity in the auditory cortex (Schiavo et al., 2020). The study showed that single-unit responses to played-back pup calls were increased during co-housing up to retrieval onset for each mouse, and for non-co-housed virgins, PVN spiking transiently increased concomitantly with enhanced cortical responses to pup calls, even before retrieving pups. The authors also found that the photometric signal increased before the retrieval onset and decreased after retrieval onset. Therefore, PVN activity was closely associated with cortical plasticity in a self-adaption way.

**Figure 1:**
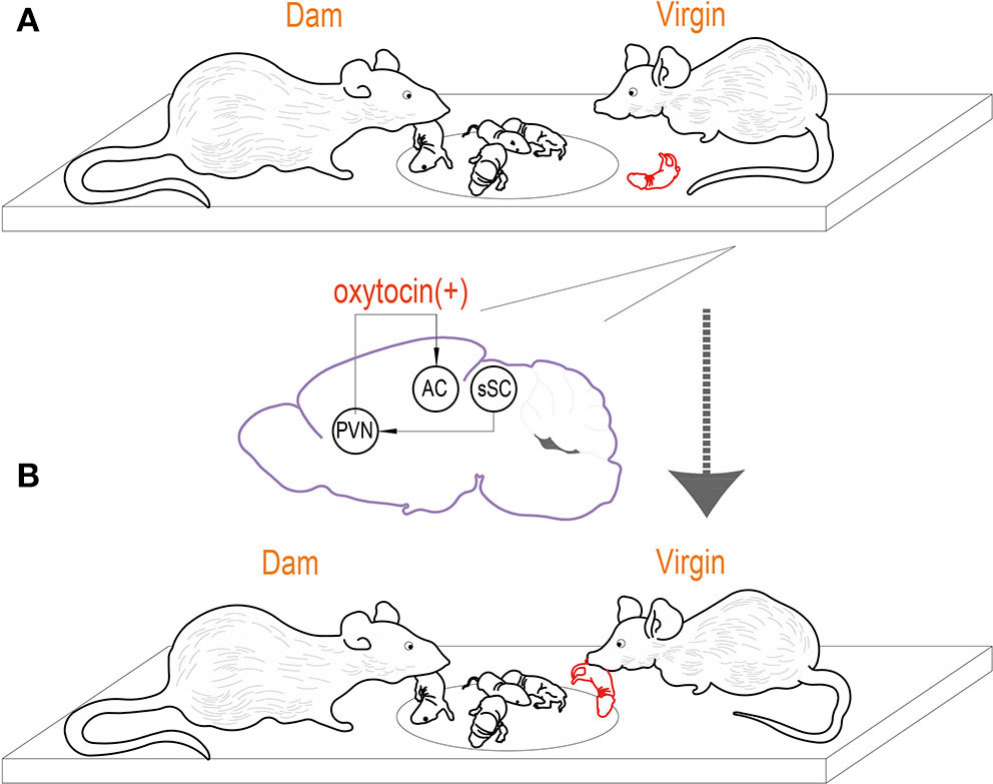
Virgin mice can learn how to raise and retrieve pups by observing. **(A)** PVN to auditory cortex projections modulated by oxytocin is necessary for the learning of pup retrieval. In virgins that begin retrieving, PVN to auditory cortex projections are activated during observation of dam retrieval, on the contrary, they are not significantly activated in virgins that did not learn after observation. **(B)** The virgin mouse start to take parenting task after observing reproductive behavior of co-housed dam.

Finally, the relationship between pup retrieval and signaling from virgin PVN to the left auditory cortex was explored. The data demonstrated that PVN to auditory cortex projections was only significantly activated in virgins when began retrieving, and the activation was related to oxytocin signaling. Selective inhibition of OT receptors in the left auditory cortex of virgin mice can prevent their retrieval of them, while the activation of OT-PVN to auditory cortical projection can enhance the retrieval of virgin mice. These results suggested that OT-PVN to auditory cortical neural projection was positively correlated with OT signal (Figure 1).

## Discussion

OT plays a fundamental role in the establishment and quality of parent-infant bonding. Large-scale and whole-brain anatomical mapping, enabled by oxytocin-Cre mice and oxytocin neuron-specific viruses, which were firstly used in rats and later in mice, revealed that oxytocinergic axons throughout the brain, including in the thalamus, cortex, amygdala, striatum, and hippocampus, have different functions after being activated (Irani et al., 2010; Knobloch et al., 2012; Mitre et al., 2016; Zhang et al., 2021). Important insights have been gained from a variety of studies into the role of OT, such as social regulation, affiliation, empathy, trust and the promotion of social behavior and the parent-child interactions (Kosfeld et al., 2005; Donaldson and Young, 2008; Gordon et al., 2010; Bartz et al., 2011; Stevens et al., 2013). Feldman and colleagues found that parental behavioral sensitivity was associated with OT production, which appears to be specific to the attachment relationship (Feldman et al., 2007). A deeper neurophysiological understanding of OT may even help parents and children to regulate stress, especially during the COVID-19 pandemic, because isolation is a significant stressor for newborn infants and could worsen the disease's progression for those previously infected with SARS-CoV-2 (Ionio et al., 2021). Hardin and colleagues reported that there were higher levels of the mother-infant dyad oxytocin in the kangaroo care group compared with the control group (Hardin et al., 2020). As available evidence showed that psychological problems in maternal separation's offspring are more significant than the potential harms of maternal skin-to-skin contact in terms of the proximity of SARS-CoV-2, skin-to-skin contact is encouraged by the WHO for all women (World Health Organization, 2021).

Alloparenting behaviors have been studied in many species. In human society, palaeoanthropological and comparative evidence suggested that enhanced allomaternal care may underpin the marked transitions in sociality, life history, and brain size of ancestral hominins (Isler and Van Schaik, 2012). A variety of studies that focused on alloparental behavior were carried out (Martin et al., 2020; Shaver et al., 2020; Finton et al., 2022) in recent decades, and a study has provided evidence of plasticity in human alloparenting in response to ecological contexts, comparable to previously observed patterns across avian and mammalian cooperative breeders (Martin et al., 2020). The findings of Froemke's team provide scientific evidence for a deeper understanding of alloparenting in mammals. Their findings offer new promising solutions for mental and psychological problems such as psychosocial stress regulation during the present COVID-19 crisis and child abuse. The COVID-19 crisis has affected and is still affecting our society at multiple levels. To avoid overloading the medical system and spreading SARS-CoV-2, repeated and long-lasting social restrictions have been implemented in many countries. However, the results of social restrictions included the loss of daily routines, financial restraints, social tension in families, social isolation and generally increased levels of psychosocial stress (Gryksa and Neumann, 2022). The pandemic-related social distancing results in substantial inactivation of the OT system in the general population. Therefore, upregulating or at least maintaining the activity of OT signaling has a profound implication to prevent or reverse social isolation/social stress-induced impairments of mental wellbeing and general health. Probiotics such as Lactobacillus bacteria were described to increase OT levels (Burkett et al., 2016). Moreover, Burkett et al. reported that oxytocin mediates empathy-based consoling behaviors in prairie voles (Bharwani et al., 2017). Could subtle social interactions, such as distant social media with visual and auditory social cues, stimulate the OT system and act as a way for social stress regulation? This needs further investigation but with great social meaning. In addition, child abuse is arguably the most poorly understood form of psychological problem, compared with physical and sexual abuse, much less is known about its presentation, causes, consequences, prevention, and treatments. Fortunately, more and more researchers are paying attention to OT (Suzuki et al., 2020; Mizuki and Fujiwara, 2021; Ramo-Fernández et al., 2021). It was reported that genetic variants of OXTR may contribute to the aggression susceptibility of mice? (Zhang et al., 2018).

The above studies provided evidence of nursing behavior comprehensively and scientifically, which combined behavior and neural activity monitoring. However, there are still several questions that need to be further addressed. First, previous studies have shown that testosterone, estradiol and prolactin may also be associated with alloparenting, it is hard to draw a conclusion that OT plays an independent role in alloparenting behavior, as complex neurophysiological processes always act as an outcome of the synergistic effect of hormones. Second, there is species heterogeneity about alloparenting (Kenkel et al., 2017), therefore, clinical research based on the results of animal experiments is necessary. In the long run, these findings built a bridge between biological research about oxytocin and future social psychological research.

## Author Contributions

XF, YW, and ZH contributed to writing and editing the manuscript. All authors contributed to the article and approved the submitted version.

## Funding

This work was supported by the Scientific Research Foundation for Scholars of HZNU (4125C5021920453 and 4125C50220204109).

## Conflict of Interest

The authors declare that the research was conducted in the absence of any commercial or financial relationships that could be construed as a potential conflict of interest.

## Publisher's Note

All claims expressed in this article are solely those of the authors and do not necessarily represent those of their affiliated organizations, or those of the publisher, the editors and the reviewers. Any product that may be evaluated in this article, or claim that may be made by its manufacturer, is not guaranteed or endorsed by the publisher.
